# Intrathecal Drug Delivery Systems for Chronic Pain Management: A Narrative Review of Pharmacologic Agents, Clinical Applications, and Considerations

**DOI:** 10.3390/pharmacy13060185

**Published:** 2025-12-16

**Authors:** Milan Patel, Alison J. Deng, Madelyn Reilly, Mariam Morcus, Alyssa McKenzie, Lukas Henjum, Alan D. Kaye, Alaa Abd-Elsayed

**Affiliations:** 1 Department of Medicine, New York Institute of Technology College of Osteopathic Medicine at Arkansas State University, Jonesboro, AR 72401, USA; mpate337@nyit.edu; 2 Department of Medicine, Loyola University Chicago Stritch School of Medicine, Maywood, IL 60153, USA; adeng1@luc.edu; 3 Department of Anesthesiology, University of Wisconsin School of Medicine and Public Health, Madison, WI 53705, USA; mjreilly2@wisc.edu (M.R.);; 4 Department of Medicine, St. Georges University, University Centre Grenada, West Indies 11739, Grenada; 5 Department of Anesthesiology, Louisiana State University School of Medicine, Shreveport, LA 71130, USA; alan.kaye@lsuhs.edu

**Keywords:** intrathecal drug delivery systems (IDDS), intrathecal pump(s), morphine, baclofen, hydromorphone, fentanyl, sufentanil, artificial intelligence

## Abstract

This narrative review seeks to delve into the different on and off-label medications commonly used with intrathecal drug delivery systems (IDDS) and their clinical applications specifically in pain management settings. This review utilizes a variety of studies including reviews, retrospective chart analyses, and more to analyze the current effectiveness of various pharmacological agents on reducing chronic pain through IDDS. The initial results of intrathecal delivery of these medications have provided benefit in pain reduction and overall patient satisfaction; however, this review will seek to analyze the current data and understanding and suggest areas of strength and improvement within the field and our current understanding.

## 1. Introduction

Chronic pain, which is defined as pain persisting for more than 3–6 months, is a leading cause of morbidity in the United States and is a common reason why adults seek medical attention [1]. While systemic pharmacotherapy such as oral opioids, antidepressants, and anticonvulsants can often provide moderate pain relief, patients may build a tolerance for a given medication, develop a dependence or addiction, or suffer adverse side effects, all of which compound to the burden of chronic pain [2]. For example, many systemic medications, especially those with a large therapeutic window, are ineffective at low doses and require higher and more frequent doses to control intense pain [3]. In particular, opioids, which often require higher doses to reach a therapeutic effect for chronic pain, since opioid receptors in the brain can become sensitized with chronic use [3]. Because many patients with chronic pain also have comorbidities and other medical diagnoses that require additional systemic medications, the risk of drug interactions is high and requires monitoring to prevent adverse outcomes [4]. Although efforts to reduce opioid prescribing for chronic pain have improved since the opioid epidemic began in the late 1990s, opioid overuse and addiction remain a public health issue in the United States and Canada and warrant further research into alternative pain control methods [5]. However, opioid prescribing is still limited in Europe. Furthermore, many patients have pain that is refractory to systemic pharmacotherapy and require other interventions and techniques to manage their pain and improve their quality of life.

Intrathecal drug delivery systems (IDDS), often referred to as pain pumps, have shown promising success for the management of various refractory chronic pain syndromes and are safe and effective methods for treating chronic pain [6]. IDDS are surgically implanted devices designed to deliver medication directly into the cerebral spinal fluid (CSF) in the intrathecal space surrounding the spinal cord [7]. In comparison to systemic therapies, IDDS utilizes lower doses of analgesics since the medication is delivered directly into CSF and bypasses the blood–brain barrier to enter the central nervous system (CNS). Additionally, the targeted delivery to the spinal cord enhances the effectiveness of the medication by allowing the drug to act on pain receptors in the intrathecal space, thus creating a more potent and immediate effect on pain relief at lower doses [8].

IDDS are becoming increasingly useful in managing pain for conditions such as chronic non-cancer pain, cancer pain, and spasticity [9]. Specifically, intrathecal pumps (ITPs) containing baclofen, a muscle relaxant and antispasmodic medication, can provide significant relief for severe spasticity caused by conditions such as cerebral palsy, spinal cord injury, and multiple sclerosis [10]. The first IDDS was implanted in a cancer patient in 1981 and provided low-dose morphine for cancer-related pain, and since then, several studies have shown that IDDS are safe and effective [11]. However, as of 2004, there are now three monotherapy drugs approved by the United States Food and Drug Administration (FDA) for use in IDDS, including morphine, ziconotide, and baclofen [12].

According to the Polyanalgesic Consensus Conference (PACC), a panel to review and improve the safety and efficacy of intrathecal drug delivery, patient selection is crucial in determining who is eligible for IDDS and includes various factors such as the complexity of the patient’s disease, comorbidities, previous failure of conservative therapies, psychological state, adherence to treatment recommendations, and surgical candidacy [13]. However, there are no universally accepted guidelines or recommendations for patient selection. Instead, patients are typically separated into categories of cancer and non-cancer-related pain [12]. Before implantation of the pump and catheter, a trial procedure is performed to assess the patient’s response to the drug. The four trialing methods include single-shot intrathecal bolus injection, multiple intrathecal bolus injections, continuous intrathecal infusion, and continuous epidural infusion, all of which have shown equivocal efficacy [14]. If the trial is successful, the intrathecal pump ITP and catheter are percutaneously or surgically implanted, and the patient must maintain close follow-up to assess post-implantation pain levels.

The use of IDDS as a treatment for chronic pain has many advantages, such as improved quality of life, personalized treatment plans, reduced side effects, and cost-effectiveness over time [11]. However, as with all procedures, there are important risks to consider. Surgical complications, device malfunction, granuloma formation, and infection are possible and must be discussed with the patient before proceeding, especially since it is an elective procedure [15]. However, the benefits typically outweigh the risks, especially for patients with pain refractory to more conservative pain management techniques.

This literature review aims to review the mechanisms, pharmacological agents, clinical applications, benefits, complications, and future directions of intrathecal drug delivery systems in the management of chronic pain.

## 2. Intrathecal Drug Delivery Systems (IDDS) Overview

The primary components of IDDS are the pump, catheter, medication reservoir, and refilling port. The pump is a small metal device that is implanted under the skin, typically in the abdominal wall or the gluteal area [16]. The pump houses the medication reservoir that delivers the medication through a catheter, a thin and flexible tube, into the intrathecal space surrounding the spinal cord [16]. The medication reservoir can be accessed percutaneously through the refilling port to change or refill medications [17].

Pumps for IDDS are available in non-programmable and programmable formats. Non-programmable pumps, also known as continuous flow pumps, deliver the medication at a fixed rate, thus allowing for a continuous and steady flow 24 h per day [18]. Non-programmable pumps utilize a gas chamber for flow generation and therefore do not rely on a battery or electrical current to operate [19]. However, because the flow is set to a constant rate, the dosage of the medication can only be changed by altering the concentration of the drug within the pumps’ reservoir, so these types of pumps are less commonly used [19]. On the other hand, programmable pumps, also known as variable flow pumps, allow for flexible medication delivery, enabling the provider to adjust the flow rate, timing, and dosage as needed. The flexibility of programmable pumps allows for a more individualized treatment plan since the provider can tailor the treatment to the patient’s specific needs, which can be particularly beneficial for fluctuating pain levels or different needs at different times of the day. Additionally, programmable pumps allow for the delivery of a bolus, which can be valuable for breakthrough pain. Programmable pumps, however, do typically rely on a battery to operate, which will need to be replaced when depleted [11]. While both types of pumps offer different advantages, it is important to consider the cost and flexibility of the pump as well as the patient’s specific pain condition, lifestyle, and response to previous treatments when deciding what type of pump to use.

Delivering medications through oral or parenteral methods can be challenging because of the blood–brain barrier, which serves as a protective filter that restricts many compounds, particularly macromolecules, from accessing the CNS [11]. Intrathecal drug delivery bypasses this barrier by introducing medications directly into the CSF, allowing them to reach critical areas such as the dorsal horn of the spinal cord without interference from the blood–brain barrier or the effects of first-pass metabolism [20]. Once the medication has been pumped through the catheter into the CSF, it diffuses and permeates across the pia mater, arachnoid, and white matter of the spinal cord to bind to opioid receptors in the dorsal horn, which are involved in nociceptive processing and the transmission of pain signals [21]. Because the longitudinal spread of the drug along the spinal cord is limited to only a few vertebral levels, precise catheter placement is crucial to dictate where the drug’s highest concentration will be, which directly affects the area of pain relief [21]. By injecting directly into the intrathecal space, the drug bypasses the bloodstream and gastrointestinal tract, ultimately minimizing systemic side effects.

Various factors influence the pharmacokinetics of CSF as well as intrathecal drug distribution. CSF is produced primarily in the choroid plexus and circulates in an oscillatory and pulsatile fashion that corresponds to heart rate throughout the ventricular and spinal subarachnoid spaces, with no overall net flow [22]. Spinal CSF movement is primarily influenced by pressure changes caused by brain-blood pulsations and by fluctuations in intrathoracic pressure resulting from diaphragm contractions during respiration [23]. As such, cerebral perfusion and venous drainage drive CSF flow, particularly within the spinal cord, through their effects on pressure gradients and pulsatile dynamics. Arterial blood entering the brain with each heartbeat generates rhythmic intracranial pressure changes that help drive CSF movement, a mechanism known as arterial pulsation-driven flow [21]. At the same time, venous drainage regulates intracranial and intraspinal pressure and maintains a steady CSF volume [21]. Together, the balance between arterial inflow and venous outflow creates a dynamic pressure environment that shapes the direction, amplitude, and rhythm of CSF movement along the craniospinal axis. Furthermore, drug metabolism and clearance within the CSF are influenced not only by CSF flow dynamics but also by injection volume and rate and drug-specific properties such as drug solubility, molecular weight, uptake kinetics, and binding affinity [24]. For example, higher injection volumes and rates can lead to faster and wider drug distribution within the CSF [24]. Understanding these dynamics is essential for optimizing drug dosing, catheter placement, and achieving targeted therapeutic effects while minimizing systemic exposure.

As previously mentioned, IDDS is indicated for non-cancer pain conditions, cancer-related pain, and severe spasticity and is specifically tailored to patients who are refractory to oral or transdermal analgesic delivery. Common non-cancer-related pain conditions that warrant the use of IDDS include complex regional pain syndrome, spondylolisthesis, spondylosis, back surgery failure syndrome, severe neuropathic pain, spinal stenosis, and compression fractures [8]. A recent comprehensive review highlights the continued evolution of IDDS for refractory chronic pain. Advances in catheter design, pump programming, and pharmacologic repertoire have improved the safety and precision of intrathecal therapies [21]. Regarding cancer-related pain, tumor characteristics, prognosis, periprocedural imaging, and the risk of disease progression need to be evaluated before implantation of the pump in oncological patients [8]. While there are many patient-specific factors to consider, some of the absolute contradictions to IDDS include coagulopathies or bleeding disorders, immunosuppression, systemic infection, active intravenous drug use, psychosis or dementia, and a life expectancy of 3 months or less [11]. Current guidelines also advise against the use of IDDS for widespread pain, headaches, or facial pain due to limited efficacy [11]. Given its indications and proven efficacy, IDDS has the ability to improve the quality of life by enhancing the physical, emotional, and psychological well-being of patients with chronic pain. According to Table 1, the primary components, pump types, and mechanisms overall can are summarized below. 

## 3. Pharmacologic Agents Used in Intrathecal Pumps

### 3.1. On-Label FDA-Approved Drugs

In accordance with the FDA, three drugs have been approved for intrathecal use. These drugs include morphine, ziconotide, and baclofen. Specifically, morphine is a great monotherapy option for both neuropathic and nociceptive pain [11]. Additionally, the synergistic action of morphine with local anesthetics, due to its distinct action, is a well-documented benefit to patients undergoing spinal anesthesia. Ziconotide is particularly indicated as a treatment for severe chronic neuropathic, cancer-related pain [25].

#### 3.1.1. Morphine

When utilizing morphine, a significant amount of morphine-targeted opioid receptors are located in the dorsal horn of the spinal cord. The binding of morphine to these receptors leads to the inhibition of neurotransmitter release, such as substance P and calcitonin gene-related peptide. Therefore, this is essentially inhibiting nociceptive transmission. When used in an intrathecal system, morphine leads to fewer unwanted side effects. This is due to morphine’s hydrophilic nature; the drug stays localized in the intrathecal space and moves throughout the spinal cord [11]. Therefore, the spread of the drug can be monitored and predicted, thus leading to fewer adverse events as compared to anesthetics. Given morphine’s hydrophilic properties, intrathecal distribution can be evaluated with contrast-based myelography or nuclear imaging studies to track flow within the CSF. These methods allow clinicians to confirm drug spread and detect issues such as impaired flow or catheter malfunction.

The dosage of morphine necessary for positive results is quite low. The greater the amount of morphine that is administered, the higher the chances of adverse events occurring. Thus, the recommended dosage of morphine is 1/300th of the individual patient’s total oral morphine dosage, with the possibility of positive results able to be achieved with even smaller amounts. Additionally, the addition of bupivacaine can increase the analgesic effects with limited risk of adverse outcomes. However, the exact mechanism of action behind this synergistic effect is not fully understood [11].

#### 3.1.2. Ziconotide

Ziconotide is also an FDA-approved drug for intrathecal usage. The drug binds to N-type voltage-sensitive calcium channels on nociceptive afferent nerves, also in the dorsal horn of the spinal cord. Consequently, this affects the release of pro-nociceptive neurotransmitters, leading to analgesic results with application in cancer-related pain such as metastatic bone pain. Ziconotide administered intrathecally does not display any adverse events or withdrawal symptoms upon interruptions of note; however, it is not completely in the clear either. In higher dosages, around 25% of patients reported symptoms of dizziness, nausea, confusion, and nystagmus. Furthermore, less than 5% of patients described more severe symptoms such as urinary retention, blurred vision, double vision, diarrhea, vomiting, constipation, limb pain, muscle spasms, ataxia, drowsiness, tremors, and altered taste. Lastly, the rarest of additional symptoms include those of a psychological nature, including anxiety, insomnia, depression, and psychosis [26,27].

Ziconotide dosage titration accuracy is important due to the small therapeutic window. The initial continuous dosage of intrathecal ziconotide should be less than or equal to 1.2 mcg/d. To minimize or reduce the chance of adverse events occurring, it is recommended that titrations increase in small increments less than or equal to 0.5 mcg/d every 4 to 7 days, with the maximum dosage being 19.2 mcg/d [11,28,29].

#### 3.1.3. Baclofen (Chronic Pain Specifically)

Baclofen administration through intrathecal methods requires titration. Beginning with a single test dose, for example, 50 mcg, the patient is then monitored for between 5 and 10 h. The dosage increases are carried out by 10% or 30% for spinal cord-originated spasticity and 5% to 15% for cerebral-origin spasticity. When considering the main adverse events of baclofen usage, the potential outcomes include sedation, excessive muscle weakness, dizziness, nausea, mental confusion, and drowsiness. However, if the medication is administered intrathecally, the chances of these adverse events occurring are decreased [30]. However, these adverse events can still occur intrathecally due to the dosage or inadvertent bolus.

In a study conducted by Hagemann et al., the researchers sought to determine the efficacy and value of intrathecal baclofen therapy for spasticity in infants and small children through retrospective analysis. Between 2007 and 2018, 17 patients under the age of 6 had pump implantations. The youngest infant was 11 months. Overall, the results were positive with complications coming in the form of two catheter dislocations, one catheter transection, and one pump infection [11,31].

### 3.2. Common Off-Label Drugs and Medications

#### 3.2.1. Hydromorphone

Hydromorphone is commonly compared to morphine in terms of treatment use. However, common advantages that hydromorphone displays include the decreased dosage necessary for the same analgesic effect with lessened side effects in comparison to morphine. Additionally, there are no known active metabolites [32,33]. Another key point is the increased lipid solubility of hydromorphone, which leads to rapid entry of this medication into the spinal cord [34,35].

In a retrospective review conducted by Anderson et al., 37 patients having chronic non-malignant pain were treated with intrathecal hydromorphone. Of note, this was after intraspinal morphine had failed. The average age of the patients was 64 +/− 12 years old, with all of them having severe cases of non-malignant pain as previously mentioned. Around 51% of patients had failed lumosacral spinal interventions, with morphine being switched to hydromorphone due to pharmacological complications. The complications manifested themselves as nausea, vomiting, pruritus, and sedation; however, they were reduced when the medication switch was made. Overall, analgesic response and pain relief were reduced by at least 25% in 16 of the patients. This study showed that hydromorphone can be used safely and has demonstrated positive results. However, one of the drawbacks of this paper is the time that has passed, as this study was conducted in 2001 [36].

#### 3.2.2. Fentanyl/Sufentanil

Use of fentanyl through long-term intraspinal infusion was analyzed through a retrospective analysis including 29 patients. Eight of the patients included were given fentanyl at a dose of 10.5 to 11.5 micrograms per day. For an average duration of 31 months, the patients given fentanyl reported having a 68% improvement in pain [37].

Furthermore, an additional study by Cherry et al. looked at the administration of fentanyl in chronic intractable anginal chest pain patients. Two of the seven patients analyzed were given intrathecal fentanyl over the duration of 2–7 years. Overall, the patients showed significant improvement in angina, with side effects being reported, including nausea, drowsiness, chronic urinary retention, increased sweating, lethargy, and depression [38].

Consequently, sufentanil was analyzed in a study conducted by Hassenbusch et al. This study assessed long-term IT infusion of morphine or sufentanil with the doses starting at 50 or 0.05 micrograms per hour, respectively. As the study was carried out, all of the patients who were started on sufentanil were switched over to morphine sulfate either due to ineffectiveness or side effects. Ten of the patients who responded well to sufentanil were continued on sufentanil infusion with the implantable pump. Overall, 6 out of 18 patients who were not on the morphine infusions were given sufentanil doses of 0.5 to 3.0 micrograms per hour. However, with initial pain relief being good, about 39% of patients did not consistently receive significant pain relief, with five of the patients receiving the sufentanil doses and the other two receiving the morphine doses [39,40].

#### 3.2.3. Bupivicaine

Bupivacaine is a local anesthetic agent that is commonly used for pain treatment. In an open-label study conducted by Sjoberg and Appelgren et al., they focused on assessing the long-term intrathecal use of morphine and bupivacaine in refractory cancer pain. About 52 patients were included in this study with an average follow-up time of 6 months, and the results were very positive. Overall, 96.2% of patients received good pain relief with the adverse effects including paraesthesia, paresis, gait impairment, urinary retention, anal sphincter disturbances, and orthostatic hypertension [41,42].

In an additional study conducted by Ntescu and Dahm et al., these researchers focused on continuous infusion of opioids and bupivacaine by externalized intrathecal catheters in long-term treatment of refractory nonmalignant pain. The style of this study was in the form of a prospective, cohort, non-randomized, consecutive trial with the dosage ranging from 0.8 to 27 mg/day. Overall, 90 patients were observed for chronic pain over the duration of 60 days. The results show that about 66% of patients received good to excellent pain relief with side effects including bradypnea, transient paresthesia, short-lasting pareses, temporary urine retention, episodic orthostatic arterial hypotension, and attempted suicide [42,43].

#### 3.2.4. Clonidine

Clonidine is another off-label medication for intrathecal usage. The current recommendation is to use this medication after a minimum of two other classes of intrathecal analgesics. Of note, the potential side effects of clonidine include hypotension, bradycardia, sedation, insomnia, nausea, confusion, and xerostomia [11]. In 1988, through observation of intrathecal clonidine usage, Esenach et al. conducted a study that used a 150 mcg dosage in hopes of reducing heat-induced hyperalgesia [44]. It was found that this was true, and future studies continued to build on this initial success.

Furthermore, in 2002, Hassenbusch et al. studied the effects of intrathecal clonidine through an implantable pump in chronic cancer patients. The study showed that 13 patients were found to have long-term success with a follow-up time on average of around 16.7 months. However, unfortunately, nine patients did not receive adequate pain relief [45].

An additional study conducted by Ackerman et al. performed a retrospective chart review including 15 patients in whom intrathecal clonidine was used. All of the patients were given a single shot with and without the infusion of clonidine. The results showed that 10 out of the 15 patients reported significant pain relief. However, 70% of the patients initially responded to clonidine alone, but when clonidine was used in conjunction with a second drug, intrathecal clonidine provided long-term benefit, providing relief up to 24 months. Thai was seen in 20% of patients who did not receive adequate pain relief from clonidine alone [46].

#### 3.2.5. Dexmedetomidine

The use of dexmedetomidine (DEX) includes it as a perioperative adjuvant. However, its use intrathecally continues to be explored. In this study conducted by Qian et al., the molecular mechanisms of the anti-nociceptive effects of dexmedetomidine were explored through a mouse model highlighting chronic constriction injury (CCI). The overall results of the study showed that DEX does provide an analgesic effect on chronic neuropathic pain [47]. Overall, more research needs to be conducted on the analgesic effects of intrathecal DEX for chronic pain relief, since this study focuses on elucidating the underlying mechanisms.

#### 3.2.6. Midazolam

The last off-label medication we will discuss is Midazolam. The antinociceptive effect of intrathecal midazolam comes from the spinal gamma-aminobutyric acid receptors. However, to analyze and observe the pain relief capabilities in patients, a prospective, open-label study was conducted by Prochazka et al. Overall, the analgesic effect was determined through a patient questionnaire upon multiple visits, with 50% pain reduction being determined to be the cutoff for positive results. The results of this study showed that between 1995 and 2010, 500 administrations were performed. In total, 227 administrations were given to 57 male patients, while on the other hand, 273 administrations were given to 69 female patients. Lastly, 81 administrations were given for chronic low back pain, while 419 administrations were given for failed back syndrome. With an average age of 50 years old, ranging from 28 to 86, the dose given ranged from 2 to 5 mg. Over the course of 9.7 weeks on average, the analgesic effect lasted, providing great pain relief. Overall, in 65% of patients, pain relief lasted 4 weeks or longer, with 13% of patients not receiving any pain reduction at all [48]. The use of intrathecal midazolam can be a useful tool in the treatment of chronic pain management, especially in situations where pharmacological management is yielding unsuccessful results. According to Table 2, our comprehensive table dives deeper into the on- and off-label drugs. 

## 4. Drug Solubility and Stability

The delivery of intrathecal medication depends on the stability of the drugs, along with their ability to dissolve in the cerebrospinal fluid (CSF) and the pump reservoir. Prolonged drug stability is necessary for reliable and predictable pain management, while reducing the risk of catheter blockages, lowering complications, and ensuring long-term safety.

### 4.1. Solubility in Cerebrospinal Fluid

The distribution properties of a drug administered within the CSF depend primarily on lipid solubility, molecular size, and ionization state. Hydrophilic opioids, such as morphine, remain in the CSF longer and promote segmental and rostral spread [47]. In contrast, lipophilic opioids, including fentanyl and sufentanil, penetrate neural tissues rapidly and produce brief, localized pain relief [48].

Higher concentration opioids, particularly morphine and hydromorphone, have been implicated in catheter-tip granuloma formation, in which elevated intrathecal opioid concentrations induce localized inflammatory masses at the catheter tip [49]. Beyond pharmacokinetics, reservoir compatibility is also critical in determining drug safety. Results from compatibility studies indicate that ziconotide mixed with morphine, administered intrathecally at 37 °C, tends to undergo degradation and experience potency loss; therefore, coadministration of these drugs in the same pump reservoir is not recommended [50]. However, while not absolutely contraindicated, degradation time should be considered before coadministering morphine with ziconotide in terms of efficacy. When assessed individually, morphine demonstrates chemical stability for up to 90 days under simulated intrathecal conditions, whereas ziconotide retains approximately more than 80% of its initial concentration for 45–60 days, depending on temperature, concentration, and formulation [50,51,52,53,54,55]. These ranges vary slightly based on different sources.

### 4.2. Chemical Stability of Intrathecal Agents

The chemical stability of substances within pump reservoirs determines both refill intervals and preservation of potency and safety. Hydrophilic opioids, such as morphine and hydromorphone, demonstrate long-term stability at both room and body temperature for up to three months, supporting their role as preferred intrathecal opioids [52]. In contrast, peptide-based agents such as ziconotide are highly sensitive to temperature and pH, remaining stable only in acidic solutions and degrading when coformulated with opioids or local anesthetics [50,53,55,56]. In most mixtures of morphine, ropivacaine, and ziconotide, degradation occurred, although some remained stable for 60 days at 37 °C [53]. Similarly, hydromorphone (15 mg/mL) combined with bupivacaine (10 mg/mL) preserved stability for three months at 37 °C, with minor discoloration attributed to hydromorphone oxidation that did not affect potency [54].

Additional factors, such as solution pH and osmolarity, influence solubility and long-term integrity. Ziconotide, for example, requires an acidic environment (pH 4–5) for stability, which restricts compatibility with many agents [55]. Clinically, ziconotide is specifically indicated for severe chronic neuropathic or cancer-related pain and is not broadly applicable to all pain etiologies [56,57].

### 4.3. Reservoir and Device Considerations

The pump environment also contributes to drug stability. Reservoir design, elastomeric seals, and catheter composition can lead to adsorption of lipophilic drugs, lowering their bioavailability [17]. However, these effects typically diminish once binding sites become saturated and rarely persist beyond the initial infusion point [17,50,56].

Programmable pumps extend the time between refills, which increases degradation risks for unstable drugs such as ziconotide [50,53]. Polytherapy is frequently employed, with combinations such as clonidine, ketamine, or dexmedetomidine mixed with opioids and local anesthetics. However, not all admixtures have established compatibility data, making adherence to published stability charts and PACC guidelines essential [56].

### 4.4. Implications

Poor solubility or instability leads to more frequent refills, higher infection risk, and unpredictable analgesia. Stable admixtures such as morphine with bupivacaine or hydromorphone with bupivacaine support prolonged refill intervals and steady pain management [54]. Conversely, unstable mixtures containing ziconotide require closer monitoring and more frequent replacement [50,53]. Instability may also contribute to catheter-tip granuloma formation, particularly when hydrophilic opioids like morphine or hydromorphone are delivered at high concentrations [51].

The successful operation of IDDS depends on drug stability and solubility. Incorporating stability-tested drug combinations, using compatibility guidelines, and exploring advances such as nanoparticle-based formulations and biosimulation models of CSF drug distribution [57] may further enhance safety, prolong device function, and optimize patient outcomes.

## 5. Side Effects and Complications

### 5.1. Drug-Specific Adverse Effects

In determining the uses and benefits of intrathecal drug delivery, it is important to consider the adverse effects of other pain management modalities. Opioids are one of the most effective pharmacologic agents for severe pain, but are associated with a number of side effects. Common side effects include sedation, dizziness, nausea, vomiting, constipation, and delayed gastric emptying. More severely, opioids can cause respiratory depression leading to hypoxia, coma, or even death [58]. Other important considerations to be factored when prescribing opioids are tolerance and physical dependence, which can lead to increased risk of overdose, warranting limiting or stopping opioid prescriptions in some cases.

Another option for pain management is ziconotide. Some side effects of ziconotide are confusion, memory impairment, hallucination, presyncopal and syncopal episodes, gait disturbances, meningitis, depression, and elevated creatinine kinase [59]. Clonidine can also be used for managing chronic pain. However, clonidine poisoning can occur at increased dosages. During the first one-and-a-half hours, loss of consciousness may occur. By 4 h, cardiovascular symptoms, including bradycardia and hypotension, may occur. Other symptoms include somnolence, respiratory depression, and miosis [60].

Some of the potential side effects associated with various local anesthetic injections, with agents such as lidocaine, include dizziness, headaches, blurred vision, twitching muscles, weakness, and numbness at the site of injection; however, these side effects are temporary. Another disadvantage of local anesthetics is that, in terms of treating chronic pain, the pain-relieving effects are transient, typically lasting from as short as 30 min to about 8 h for long-acting agents, such as bupivacaine. An alternative option for local anesthesia is peripheral nerve blocks, which can be used for acute pain in the context of procedural or perioperative analgesia or for long-term management of chronic pain. Risks associated with peripheral nerve blocks include nerve injuries, infections, rashes, pruritus, and bleeding.

Polypharmacy has been shown to improve chronic pain, but is also associated with some side effects. For instance, in about 30% cases of severe chronic pain, opioids are used in conjunction with smooth muscle relaxants, which have been shown to further increase risks of overdose [61]. Furthermore, peripheral nerve blocks can be combined with corticosteroids (i.e., bupivacaine and dexamethasone) in order to prolong their efficacy. Despite the benefits of the prolonged analgesia, some patients were reported to have sensory and motor blockades for up to 42 h, which can hinder patients’ functionality [62]. The effects of polypharmacy also depend on the number of medications used and the ages of the patients. In geriatric patients, polypharmacy has been associated with increased frailty, increased fall risks, cognitive impairment, physical impairment, and, in some cases, death [63].

### 5.2. Mechanical and Infectious Complications

Since the placement of the intrathecal drug pump is an invasive procedure, there are risks of hemodynamic instability and infection. As implantation is an invasive surgical procedure, it carries a risk of postoperative infection that can stem from contamination, wound creation, and hardware placement. Hemodynamic instability may also occur during the procedure, most often as a result of sedation, positioning, or underlying comorbidities. After the device is implanted, hemodynamic instability may be a result of pump malfunctions or unintended bolus release. Abrupt stoppage of drug delivery due to pump malfunction can cause symptoms such as tachycardia and hypertension, depending on the drug being delivered [63]. The insertion of the catheter is also associated with complications, including migration of the catheter, laceration, occlusion, or disconnection. Another major complication associated with the insertion of the intrathecal pumps is catheter-tip-associated granulomas (CTG), which are caused by an inflammatory response and can lead to irreversible neurological damage, worsened pain, or progressive paraplegia [63].

There are also risks that are associated with the programming and functionality of the pump. For example, older pump generations are more likely to corrode. Additionally, pumps rely on electricity or batteries, so if there is an interruption in the power source, this can cause the pump to fail.

Additionally, there are risks associated with human error in regard to dosage adjustments, pump refilling errors, and risking complications secondary to potential overdoses. Side effects vary greatly depending on the respective medications, but can range from nausea and dizziness to neurocognitive deficits, respiratory depression, and death [12]. Furthermore, if patients are undergoing an MRI, the magnetic fields could temporarily hinder functionality leading to pump failure [15]. However, the pumps should be programmed in MRI before the patient undergoes MRI to minimize this complication.

## 6. Patient Selection and Trialing

It is important to consider the patients participating in a trial, especially whether their pain is cancer-related. Currently, cancer pain is considered a category of chronic pain conditions in the International Classification of Diseases version 11 (ICD-11) [64,65]. For patients with cancer, infusion therapy is indicated in patients receiving consistent narcotic therapy with no pain relief, including following escalating doses. Additionally, the tumor should not be encroaching on the thecal sac, and the patient’s life expectancy should be more than three months [66]. There are additional considerations for patients undergoing concurrent treatments or with comorbidities [67]. Pain reduction expectations in studies are different based on whether the pain is cancer-related, including in one study where, for non-cancer pain, the expectation was at least six months of relief versus three months of relief if the patient was experiencing cancer-related pain [68]. Patient selection for intrathecal therapy is more stringent for non-cancer-associated pain and will include trialing [37].

Psychological screening is important to conduct, especially in patients with noncancer pain. Its importance was first recognized in 1985 when researchers found that psychological assessments predicted pain relief from electrode implantation [69]. It is widely accepted that mental and physical health are linked. Holistic care includes understanding an individual’s barriers and needs [70]. Therefore, the Polyanalgesic Consensus Conference (PACC) recommends psychological evaluation for patients prior to undergoing intrathecal drug delivery. Furthermore, the PACC recommends periodically reassessing psychological needs and providing necessary support while a patient undergoes intrathecal therapy [71]. Psychological assessment is not required in patients with cancer and other terminal conditions [71]. While PACC attempts to standardize trialing, it is notable that there are some considerations surrounding intrathecal delivery. There is variability in the guidelines based on the patients’ specific conditions, such as their type of pain and tolerance, which adds complexity to determining a standard protocol. Furthermore, PACC in 2012 acknowledged that trialing was debatable in patients with cancer-related pain, revising their previous statement [12]. Differences between recommendations and individualized recommendations introduce some considerations and limitations of IDDS.

Trialing is an important process in which medication is delivered to the spinal cord to assess its tolerability and efficacy before committing to a long-term pump [11]. The medication can be delivered either as a single shot or a short-term infusion. A single bolus injection or multiple intermittent boluses can be administered into the intrathecal space. Additionally, a provider can place a temporary catheter, and medication is continuously infused [72]. In each of these administration routes, the patient is monitored for side effects and pain levels. Medication is deemed successful if it can reduce pain by 50% with no side effects [73]. Trialing in patients with cancer is controversial and is optional in these patients [74]. Decisions should be made based on provider preference, patient considerations, and insurance requirements. While trialing may help with insurance authorization and assessment of tolerability and efficacy, it delays treatment and necessitates the discontinuation of anticoagulants [74].

## 7. Clinical Outcomes and Evidence

IDDS therapies are well-established for chronic pain. However, IDDS efficacy differs by indication. Van Zundert and Rauck provide an up-to-date narrative overview of IDDS for cancer and noncancer pain. The authors conclude that IDDS is strongly supported for refractory cancer pain, whereas evidence for non-cancer pain remains limited and warrants a more conservative approach [75]. In patients with cancer, there is strong evidence to support the use of IDDS. In a randomized controlled trial including 202 patients comparing IDDS versus comprehensive medical management, IDDS was found to improve pain control and survival in patients with cancer-related pain [76]. Furthermore, a 2022 systematic review and meta-analysis found IDDS to be effective in reducing pain for patients with cancer [77]. Another narrative review in 2018 found that recent prospective studies support the efficacy and cost-effectiveness of IDDS [78]. On the other hand, IDDS evidence is less robust for failed back surgery syndrome (FBSS). Literature on IDDS for FBSS consists primarily of observational studies, which have found some reduction in pain, although patients did not report a greater quality of life [79]. There are also great considerations for cost-effectiveness. In cases of FBSS, neuromodulation techniques such as spinal cord stimulation are recommended for long-term outcomes [80].

In a recent 2025 retrospective cohort study, researchers found that intrathecal clonidine delivery for patients with cancer pain did not have a significant change in visual analog scale pain scores or morphine milligram equivalents [81]. They found that higher dosages of clonidine lead to fewer reported side effects. The authors concluded that intrathecal clonidine may be beneficial for reducing side effects. However, these conclusions are limited in scope by their small sample size of 18 patients and wide variation between individuals. There is no comparison group either, so the results are not generalizable or confirmatory.

Another study found that high-cervical intrathecal drug delivery reduced average pain scores for nine patients facing refractory non-cancer neuropathic craniofacial pain [82]. The study’s limitations are similar to the previous one, as it has limited generalizability from the small sample size. The retrospective design also introduces selection and reporting bias.

One study found that morphine sulfate-clonidine and sufentanil-clonidine mixtures remained chemically stable for several days in syringes, supporting their practical use [83]. These results are clinically relevant in providing timely and practical knowledge on effective drugs. Nonetheless, the results are narrow in scope as they only provide evidence on chemical stability and do not address other aspects of logistics, such as sterility or in vivo bioactivity.

Currently, there is limited evidence available for understanding long-term outcomes and quality of life. A longitudinal study of 20 patients found improvements in quality of life and pain intensity in patients with non-cancer-related pain after four years [84]. However, this study was limited to a small number of patients and used self-reported data. Another study on long-term outcomes using IDDS for complex regional pain syndrome in 2019 found no considerable pain score decrease [85]. It suggests that intrathecal drug delivery may have limited long-term value for treating complex regional pain syndrome. This study was conducted on a small sample size of 26 patients. There was also heterogeneity in patient dosages and medications that limited the study’s generalizability. Furthermore, studies on cost-effectiveness yield mixed results [85]. One systematic review reported one study with conservative estimates showing that IDDS was not cost-effective, while seven others demonstrated that IDDS was cost-effective or cost-saving [84]. There is an initial high cost of the implantable device, with one study in 2013 noting a cost of $17,317 in the first year [86]. However, the same study found that the break-even point is after two years. Other studies noted recuperation costs at the 28-month, 22-month, and 4.5-month marks [87,88,89]. These estimates varied widely, while generally the evidence supports the long-term cost-effectiveness of IDDS.

## 8. Controversies and Evolving Practices

Opioid-induced hyperalgesia is a paradox in which exposure to opioids makes the nervous system more sensitive to pain stimuli [90,91]. A meta-analysis of 26 articles found that chronic opioid exposure led to decreased pain tolerance to noxious thermal stimuli, but no evidence of difference in response to electrical stimuli [92]. A major benefit of IDDS is that it can mitigate some of these effects as patients need lower doses of opioids to achieve pain relief due to direct delivery and the availability of other non-opioid options such as ziconotide [21]. However, there are case reports of opioid-induced hyperalgesia after delivery of morphine or fentanyl even with IDDS [92,93,94,95]. Thus, there is evidence to suggest that opioid-induced hyperalgesia remains a concern in IDDS.

Both low flow and intermittent timed boluses are available to deliver a constant flow of a fixed volume of drugs and pulses of medications, respectively [19]. Intermittent timed boluses pose a particular risk of complications as they deliver a higher dosage at each bolus compared to the amount delivered during constant flow. These complications include granulomas that can often be mitigated with lower dosages [21]. Complications can be worse for drugs with a shorter half-life since a dose is degraded at a faster rate. A retrospective study of 13 patients who developed catheter-tip granulomas during intrathecal therapy found that granuloma formulation was associated with increased concentrations of intrathecal morphine, providing further evidence for a dose-dependent response [96]. One case report found a second granuloma reported after switching to low-dose fentanyl and bupivacaine [97]. Thus, there are considerations for granulomas even with regimens that are not considered high risk.

Another area where more research is needed is the practice of polypharmacy. Much of the evidence base for the practice of polypharmacy is limited to observational studies and does not enable a formulation of standardized dosing [98]. Developing these guidelines is a more complicated process due to drug–drug interactions and the increased risk for side effects. PACC guidelines emphasize a monotherapy first approach and add therapies if there is not an adequate response to the first-line agents [14].

Current IDDS guidelines do not include the delivery of cannabinoids, and there are no approved intrathecal cannabinoid therapies. There is some pre-clinical evidence in mouse neuropathic pain models that intrathecal delivery of the cannabinoids tetrahydrocannabinol and cannabidiol can be a safe and effective treatment [99]. While this is promising, there is a lack of clinical and translational studies. More research is needed on their safety for intrathecal delivery and potential side effects.

PACC updated its guidelines for IDDS treatment recently in 2024, with the last update being in 2017 [21]. In 2024, PACC split guidelines based on cancer and non-cancer pain [14,100]. The guidelines for cancer-related pain include oncology-specific pharmacology and comorbidity considerations [100]. The non-cancer pain guidelines emphasize initiating therapy at low ziconotide doses and attempting opioid reduction. For cancer-related pain, the initial morphine dosage may be slightly higher. Minimization of dose is recommended to reduce complications such as granulomas [14]. The British Pain Society also released complementary guidelines on IDDS in 2024 [101]. New guidelines are released every few years to encompass the growing body of literature surrounding IDDS.

## 9. Conclusions and Future Directions

The usage of IDDS has shaped how physicians manage chronic, refractory pain, with statistics indicating that the utilization of IDDS has reduced the need for oral pain medication by approximately 50% [102]. Expanding the usage of IDDS allows for more personalized pain management regimens that promote individualized patient care. This is incredibly important for the management of complex diagnoses such as chronic pain, since one’s perception of pain is a very individual experience, dictated by a variety of factors such as genetic pain biomarkers that vary greatly between different patients.

Integrating artificial intelligence (AI) into the evolving technology behind IDDS can contribute to this. For example, since one’s experience of pain depends on specific molecules, gene expression patterns, or indicators that affect inflammation, tissue damage, and neural changes in the body, AI can be utilized to analyze patient data and develop personal treatment plans, while intentionally reducing side effects, increasing patient interactivity, and improving treatments and patients’ quality of life [102]. In addition to analyzing patients’ genetic indicators for pain, AI can also be used in physical and chemical pain sensors by analyzing metrics such as blood pressure, muscle activity, brain signals, and chemical components in sweat [102]. This knowledge can be used to alter the programming of the IDDS in order to allow for adaptive dosing.

Technology can also assist in inter-site data collection and sharing. Some practice areas have smaller patient populations or lower diversity in patient populations, in which it is more difficult to have significant research power. With the assistance of technology, sites can share data to achieve larger sample sizes and greater statistical power for research and development, helping to advance the field [102].

Additionally, surgical intervention, including reconstructive surgery, orthopedic surgery, or neurosurgery, can be considered as a definitive resolution for debilitating, chronic, medication-resistant pain that severely impacts patients’ quality of life [102]. Advancing IDDS can potentially reduce the need for pain-relieving surgery, therefore mitigating some of the inherent complications of surgery, including hemodynamic instability and infection. Alternatively, in cases that require surgery, IDDS can also be used in the management of chronic post-operative pain, which is an element that can be explored further in future research.

Recent research has also shown that the efficacy of drugs administered through IDDS and the degree of tissue penetration are affected by how they are injected as well as the patient’s body position during the time of injection [23]. Notably, this can also potentially impact patients’ side effects and risks for drug toxicity in response to the administered medication. This implies that the precision with which physicians are able to insert the IDDS can affect the effectiveness of the drug, which can also be explored in further research.

## Figures and Tables

**Table 1:** Comprehensive table describing the key parts of IDDS, selection criteria, and CSF dynamics.

Category	Key Information
Primary Components	Pump: Implanted under skin (abdomen or gluteal area); houses reservoir and controls drug delivery. Catheter: A flexible tube that delivers medication into the intrathecal space (surrounding the spinal cord). Medication Reservoir: Stores the drug; accessible via refilling port for percutaneous refills.
Pump Types	Non-Programmable (Continuous Flow): Delivers drug at a constant rate (24 h/day). Flow generated by gas chamber (no battery). Dose changed only by altering drug concentration. Less flexible, used less commonly.
	Programmable (Variable Flow): Adjustable rate, timing, and dosage. Allows bolus delivery for breakthrough pain. Provides individualized therapy for fluctuating pain. Requires battery replacement when depleted.
Clinical Considerations in Pump Selection	Consider cost, flexibility, pain condition, patient lifestyle, and response to prior treatments.
Mechanism and Rationale for Intrathecal Delivery	Bypasses the blood–brain barrier, allowing direct drug access to CSF and spinal receptors. Avoids first-pass metabolism and systemic side effects. Drug diffuses through pia mater, arachnoid, and spinal white matter to reach opioid receptors in the dorsal horn (nociceptive modulation). Precise catheter placement critical—drug spread limited to a few vertebral levels.
CSF Dynamics and Drug Distribution	CSF Production: Mainly in the choroid plexus. Movement: Oscillatory and pulsatile, driven by arterial pulsations and respiratory intrathoracic pressure changes—no net flow. Flow Drivers: Arterial inflow → intracranial pressure pulsations. Venous drainage → pressure regulation. Drug Distribution Factors: Injection volume/rate (↑ volume → wider spread). Drug properties: solubility, molecular weight, binding affinity, uptake kinetics. CSF flow dynamics determine distribution and clearance.
Indications	Non-cancer Pain: Complex regional pain syndrome, spondylolisthesis, spondylosis, failed back surgery syndrome, neuropathic pain, spinal stenosis, compression fractures. Cancer Pain: Refractory cancer-related pain after evaluating tumor characteristics, prognosis, imaging, and disease progression risk. Severe Spasticity: Unresponsive to oral/transdermal therapy.
Contraindications	Absolute: Coagulopathy/bleeding disorders Immunosuppression Systemic infection Active IV drug use Psychosis or dementia Life expectancy < 3 months
	Relative/Guideline-Based: Widespread pain Headache or facial pain (limited efficacy).
Clinical Benefits	Enhanced targeted pain relief. Reduced systemic side effects. Improved physical, emotional, and psychological well-being. Better quality of life for refractory chronic pain patients.

**Table 2:** Comprehensive table outlining the different FDA-approved and off-label drugs used with IDDS.

Drug	FDA Status	Mechanism of Action	Primary Indications	Dosage/Titration	Advantages/Key Findings	Adverse Effects/Limitations	References
Morphine	On-Label	Binds μ-opioid receptors in the dorsal horn → inhibits the release of substance P and CGRP → ↓ nociceptive transmission	Neuropathic and nociceptive pain; cancer pain	~1/300th of oral dose (very low doses effective); can combine with bupivacaine	Predictable intrathecal spread due to hydrophilic nature → localized effect, reduced systemic side effects; synergistic with local anesthetics due to distinct nature	Nausea, pruritus, sedation at higher doses; risk ↑ with high infusion rates	[11]
Ziconotide	On-Label	Blocks N-type voltage-sensitive Ca^2+^ channels on dorsal horn afferents → ↓ release of pro-nociceptive neurotransmitters	Severe chronic neuropathic and cancer pain	Start ≤ 1.2 mcg/day, titrate ≤ 0.5 mcg/day every 4–7 days; max 19.2 mcg/day	Non-opioid analgesic, no withdrawal or tolerance	Dizziness, nausea, confusion, nystagmus (~25%); severe: urinary retention, visual changes, tremors; rare psychosis or depression	[28,29,30,31]
Baclofen	On-Label	GABA-B receptor agonist → ↓ excitatory neurotransmission in the spinal cord → muscle relaxation	Spasticity (spinal or cerebral origin); chronic pain	Test dose (e.g., 50 mcg) → titrate by 5–30% depending on spasticity type	Effective for spasticity refractory to oral therapy; fewer systemic side effects intrathecally	Sedation, weakness, dizziness, confusion; risk ↑ with pump malfunction	[11,32,33]
Hydromorphone	Off-Label	μ-opioid receptor agonist (similar to morphine)	Chronic nonmalignant pain; post-morphine failure	Lower dose needed vs. morphine; study: 37 pts with morphine failure	Less nausea, vomiting, pruritus, sedation vs. morphine; no active metabolites; more lipid-soluble → rapid CNS entry	Limited study data; 2001 trial; general opioid risks remain	[34,35,36,37,38]
Fentanyl	Off-Label	Potent μ-opioid receptor agonist; highly lipid-soluble → rapid spinal entry	Chronic pain, refractory angina	10.5–11.5 mcg/day (31 months avg.)	68% improvement in pain in long-term study	Nausea, drowsiness, urinary retention, sweating, depression	[39,40]
Sufentanil	Off-Label	Potent μ-opioid receptor agonist	Chronic pain, cancer pain	0.05–3 mcg/h (variable)	Initially good relief; some maintained benefit	~39% lost benefit over time; frequent switch back to morphine	[41,42]
Bupivicaine	Off-Label	Na^+^ channel blocker → blocks nerve conduction	Cancer pain; chronic nonmalignant pain	0.8–27 mg/day continuous infusion (varies by study)	Strong synergy with opioids (esp. morphine); long-term pain control in >90% of patients	Paresthesia, paresis, urinary retention, orthostatic hypotension, gait impairment	[43,44,45]
Clonidine	Off-Label	α_2_-adrenergic agonist → inhibits norepinephrine release → ↓ sympathetic tone and pain transmission	Adjunct to opioids/local anesthetics in chronic pain and cancer pain	150 mcg test dose; often used in combination with ≥2 other agents	Synergistic with opioids; reduces hyperalgesia	Hypotension, bradycardia, sedation, insomnia, xerostomia, and confusion	[11,46,47,48]
Dexmedetomidine	Off-Label	α_2_-adrenergic agonist; modulates spinal nociceptive transmission	Neuropathic pain (investigational)	Preclinical: dosage variable (mouse CCI model)	Analgesic effects demonstrated in animal models; ongoing research	Human data limited; safety/efficacy not yet established	[49]
Midazolam	Off-Label	GABA-A receptor agonist → spinal inhibitory modulation	Chronic low back pain, failed back syndrome	2–5 mg per dose; avg. analgesia 9.7 weeks	65% had ≥4 weeks pain relief; effective for refractory cases	Drowsiness, variable response (13% no relief)	[49]

## Data Availability

No new data were created or analyzed in this study. Data sharing is not applicable to this article.
